# Ultra-high field imaging, plasma markers and autopsy data uncover a specific rostral locus coeruleus vulnerability to hyperphosphorylated tau

**DOI:** 10.1038/s41380-023-02041-y

**Published:** 2023-04-05

**Authors:** Maxime Van Egroo, Joost M. Riphagen, Nicholas J. Ashton, Shorena Janelidze, Reisa A. Sperling, Keith A. Johnson, Hyun-Sik Yang, David A. Bennett, Kaj Blennow, Oskar Hansson, Henrik Zetterberg, Heidi I. L. Jacobs

**Affiliations:** 1 Faculty of Health, Medicine and Life Sciences, School for Mental Health and Neuroscience, Alzheimer Centre Limburg, Maastricht University, Maastricht, The Netherlands; 2 Gordon Center for Medical Imaging, Department of Radiology, Massachusetts General Hospital, Boston, MA USA; 3 Harvard Medical School, Boston, MA USA; 4 Department of Psychiatry and Neurochemistry, Institute of Neuroscience and Physiology, The Sahlgrenska Academy at the University of Gothenburg, Mölndal, Sweden; 5 Centre for Age-Related Medicine, Stavanger University Hospital, Stavanger, Norway; 6 King’s College London, Institute of Psychiatry, Psychology and Neuroscience, Maurice Wohl Institute Clinical Neuroscience Institute, London, UK; 7 NIHR Biomedical Research Centre for Mental Health and Biomedical Research Unit for Dementia at South London and Maudsley NHS Foundation, London, UK; 8 Clinical Memory Research Unit, Department of Clinical Sciences Malmö, Lund University, Lund, Sweden; 9 Center for Alzheimer Research and Treatment, Department of Neurology, Brigham and Women’s Hospital, Boston, MA USA; 10 Department of Neurology, Massachusetts General Hospital, Boston, MA USA; 11 Department of Neurological Sciences, Rush Alzheimer’s Disease Center, Rush University Medical Center, Chicago, IL 60612 USA; 12 Clinical Neurochemistry Laboratory, Sahlgrenska University Hospital, Mölndal, Sweden; 13 Memory Clinic, Skåne University Hospital, Malmö, Sweden; 14 Department of Neurodegenerative Disease, UCL Institute of Neurology, Queen Square, London, UK; 15 UK Dementia Research Institute at UCL, London, UK; 16 Hong Kong Center for Neurodegenerative Diseases, Clear Water Bay, Hong Kong, China

**Keywords:** Biomarkers, Neuroscience

## Abstract

Autopsy data indicate that the locus coeruleus (LC) is one of the first sites in the brain to accumulate hyperphosphorylated tau pathology, with the rostral part possibly being more vulnerable in the earlier stages of the disease. Taking advantage of recent developments in ultra-high field (7 T) imaging, we investigated whether imaging measures of the LC also reveal a specific anatomic correlation with tau using novel plasma biomarkers of different species of hyperphosphorylated tau, how early in adulthood these associations can be detected and if are associated with worse cognitive performance. To validate the anatomic correlations, we tested if a rostro-caudal gradient in tau pathology is also detected at autopsy in data from the Rush Memory and Aging Project (MAP). We found that higher plasma measures of phosphorylated tau, in particular ptau_231_, correlated negatively with dorso-rostral LC integrity, whereas correlations for neurodegenerative plasma markers (neurofilament light, total tau) were scattered throughout the LC including middle to caudal sections. In contrast, the plasma Aβ_42/40_ ratio, associated with brain amyloidosis, did not correlate with LC integrity. These findings were specific to the rostral LC and not observed when using the entire LC or the hippocampus. Furthermore, in the MAP data, we observed higher rostral than caudal tangle density in the LC, independent of the disease stage. The in vivo LC-phosphorylated tau correlations became significant from midlife, with the earliest effect for ptau_231_, starting at about age 55. Finally, interactions between lower rostral LC integrity and higher ptau_231_ concentrations predicted lower cognitive performance. Together, these findings demonstrate a specific rostral vulnerability to early phosphorylated tau species that can be detected with dedicated magnetic resonance imaging measures, highlighting the promise of LC imaging as an early marker of AD-related processes.

## Introduction

Alzheimer’s disease (AD) pathogenesis starts two to three decades before the emergence of clinical symptoms [1]. Recognition of this protracted evolvement of the disease along with the observation that clinical trials targeting patients in the prodromal or later stages of the disease have not successfully resulted in functional changes, underscore the importance of detecting and implementing interventions for AD at a much earlier asymptomatic stage [2, 3]. In the search for the earliest and meaningful markers of AD-related changes, the brainstem locus coeruleus (LC) has attained significant attention [4].

Autopsy studies reported accumulation of hyperphosphorylated tau in the LC starting as early as age 20 [5, 6]. Detecting tau in the LC using current positron emission tomography (PET) ligands is arduous given the off-target binding to neuromelanin cells that also accumulate in the LC [7–9]. But, developments in MRI methods have enabled the localization of the LC in vivo [10], and work by our group indicated that the LC MRI-signal can convey information related to tau and risk of AD [11, 12]. We demonstrated that lower MRI-based LC integrity measures correlate with greater tau deposition in early cortical regions in clinically normal individuals starting approximately 54 years of age. In addition, at subthreshold levels of β-amyloid (Aβ)-PET, we observed a steeper decline in memory for individuals with lower LC integrity as compared to those with higher LC integrity, demonstrating a synergistic effect of LC integrity and early AD pathology on downstream clinical symptoms [11].

Importantly, postmortem work indicated that the rostral and middle sections of the LC are possibly susceptible to volume loss at an earlier stage in the disease than the caudal section of the LC [13]. Because the LC does not undergo significant neuronal loss before Braak stage IV [13], this suggests that these volumetric differences may be tau related [14]. Accordingly, recent 3 T MRI studies also reported that lower integrity in the rostral section of the LC correlated with memory decline, lower cortical thickness and greater risk of mild cognitive impairment (MCI) [15–17]. However, there is inconsistent data on the relationship between tau-PET and regional LC integrity. A study combining data from healthy controls, MCI and AD patients, showed that lower middle-caudal LC integrity was associated with greater temporal lobe MK-6240 tau-PET burden [18]. On the other hand, lower rostral-middle LC integrity in a group of autosomal dominant Alzheimer’s disease (ADAD) patients (symptomatic and asymptomatic) was associated with greater occipito-temporo-parietal FTP-PET burden [19]. These inconsistencies may be due to the merging of distinct disease stages, a more accelerated disease progression in ADAD [12], or the use of different tau-tracers. Even though MK-6240 is presumed to signal earlier tau aggregations than the FTP tracer, both tracers are less sensitive to earlier non-fibrillar forms of tau [20].

New advances in fluid biomarkers can act as proxies for soluble tau originating from the central nervous system, ushering a new era for the early detection of AD. Based on postmortem and the available imaging data, we hypothesized that specifically the rostral part of the LC would be vulnerable to early AD tau-related processes. This hypothesis can now be evaluated by taking advantage of exciting developments in fluid biomarkers using high sensitivity immunoassay technologies that resulted in several well-validated and robust plasma markers of neurodegeneration (neurofilament light (NfL), total tau) [21, 22], β-amyloid (Aβ) and various hyperphosphorylated tau (ptau) species [23]. Plasma ptau epitopes (ptau_181_, ptau_217_, ptau_231_) levels are concordant with autopsy findings, have high sensitivity and specificity to detect tau-pathology on PET, and increase early in the preclinical stage of AD [24–27]. In particular, ptau_231_ associated more robustly with tau-PET in asymptomatic individuals and was able to differentiate PET Braak stage 0 from Braak stage I/II and detect incipient amyloid pathology [28–30].

To examine regional vulnerability within the LC, it is important to consider that 3 T MRI methods acquire the LC in anisotropic voxels size (i.e. 0.3x0.3x3mm), which are often resampled to isotropic resolution. Such procedures can introduce noise and distortions in small regions such as the LC. Our efforts in 7 T MRI now provide a unique, detailed window in localization and anatomy of the LC at 0.4 mm near-isotropic voxel size [31, 32]. Here, we related LC integrity using 7 T MRI to different AD plasma markers in asymptomatic individuals across a wide age range, including individuals with preclinical AD. We then examined whether LC integrity and plasma markers are interactively or independently associated with cognition. To validate our in vivo findings, we also compared the proportion of neurons that were tau-positive in the rostral LC versus those in the caudal LC in 77 autopsy cases from the Rush Memory and Aging Project (MAP).

## Materials and methods

### Participants

#### The 7T dataset

Cognitively unimpaired individuals (age range = 30–85, 52 females (52.50%)) were recruited from the general community in the most Southern region of the Netherlands. All participants were screened to exclude a history of major psychiatric or neurological disorders, having a history of brain injury of brain surgery, taking medications that may influence cognitive functioning, or being not eligible for ultra-high field MRI-scanning. To exclude individuals with depressive symptoms, we screened all participants using the Hamilton Rating Scale for Depression (all within normal range = 0–12; mean  ±  SD = 2.23  ±  2.53). All participants received monetary compensation and provided written informed consent. Approval of the experimental protocol was obtained from the local ethical committee of the Faculty of Health, Medicine and Life Sciences at Maastricht University, The Netherlands.

#### The MAP dataset

The dataset included 77 participants from the Rush Memory and Aging Project (MAP), an ongoing longitudinal clinical-pathologic studies that started in 1997 [33, 34]. Eligibility criteria included age>55 years, absence of a previous dementia diagnosis and consent to annual clinical evaluation and brain autopsy at death. Participants were recruited from retirement communities, social service agencies and subsidized housing facilities, and individual homes in the Chicago metropolitan region. This sample included individuals for whom detailed LC neuropathology data was available and consisted of individuals with normal cognition (*n* = 29), mild cognitive impairment (MCI) (*n* = 27) or AD (*n* = 21) at their last clinical visit prior to autopsy. Diagnosis was done each year by a neuropsychologist and clinician, and final diagnosis was by a neurologist blinded to postmortem data, based on the National Institute of Neurological and Communicative Disorders and Stroke and the AD and Related Disorders Association (NINCDS/ADRDA) criteria [35–37].The average time interval between last visit and death for these participants was 0.77years (SD = 0.60). All data were de-identified and shared with a Data User Agreement. The study was approved by an institutional review board of Rush University Medical Center. All participants signed an informed consent, an Anatomical Gift Act, and a repository consent which allowed their data to be shared.

### Structural magnetic resonance imaging (7 T dataset)

#### MRI data acquisition

MR scans were performed in a 7 T Magnetom Siemens (Siemens Healthineers, Erlangen, Germany) with a 32-channel head coil (Nova Medical, Wilmington, MA, USA). First, we acquired a Magnetization Prepared 2 Rapid Acquisition Gradient Echoes (MP2RAGE) sequence [38] for whole brain imaging (TR = 5000 ms, TE = 2.47 ms, flip angle = 5^o^/3^o^, voxel size = 0.7 × 0.7 × 0.7 mm^3^, number of slices = 240). An in-house developed magnetization transfer-weighted turbo flash (MT-TFL) sequence [31] was performed to image the LC at high resolution. The sequence consisted of a multi-shot 3D-readout (TR = 538 ms, TE = 4.08, flip angle=8^o^, voxel size=0.4 × 0.4 × 0.5 mm^3^, number of slices = 60) with center-out k-space sampling, preceded by 20 long off-resonant Gaussian sinc pulses (pulse length = 5.12 ms, bandwidth = 250 Hz, B_1_ = 0.25 μT). For the MT-TFL sequence, the field-of-view was placed perpendicular to the pons and covered the area between the inferior colliculus and the inferior border of the pons.

#### MRI data processing

The MP2RAGE images were processed with FreeSurfer v6.0.0 (https://surfer.nmr.mgh.harvard.edu) using the software package’s automated reconstruction protocol as described previously, including expert options for 7 T images [39]. Briefly, each T1-weighted image was subjected to an automated segmentation process involving intensity normalization, skull stripping, segregating left and right hemispheres, removing brainstem and cerebellum, correcting topology defects, defining the borders between gray/white matter and gray/cerebrospinal fluid, and parcellating cortical and subcortical areas. We visually inspected and, if necessary, edited each image. As a control measure for the LC, we extracted the bilateral hippocampal volume and adjusted it for intracranial volume (eTIV) using the following equation [40]:

$${{{{{{{\mathrm{Adjusted}}}}}}}}\;{{{{{{{\mathrm{hippocampal}}}}}}}}\;{{{{{{{\mathrm{volume}}}}}}}} = 	\, {{{{{{{\mathrm{raw}}}}}}}}\;{{{{{{{\mathrm{hippocampal}}}}}}}}\;{{{{{{{\mathrm{volume}}}}}}}}\\ 	- b\left( {{{{{{{{\mathrm{eTIV - Mean}}}}}}}}\;{{{{{{{\mathrm{eTIV}}}}}}}}} \right)$$

where *b* indicates the regression coefficient when raw hippocampal volume is regressed against eTIV.

The MT-TFL images were intensity-normalized in a slice-specific manner using the subject-specific mean intensity of a 10 × 10voxel region-of-interest located in the pontine tegmentum (PT). From these images, a study-specific template of the LC scans was created with an iterative diffeomorphic warp estimate using the buildtemplateparallel.sh script of ANTS, as described previously [32, 41]. The LC was manually delineated on the common space twice by an expert (HJ, spatial correlation r = 0.90) and by another rater (spatial correlation between raters r = 0.83), guided by the voxel intensities and known LC anatomy. This segmented LC mask was applied to the high-resolution individual spatial and intensity-normalized LC images (Supplemental Fig. 1). From the template we constructed a surface rendering for visualization purposes of our results. To show regional specificity, we also extracted intensity values from the entire LC. Using the inverse warp, we further obtained subject-specific LC segmentations, extracted the bilateral LC volume and adjusted it for eTIV for control analyses.

### Plasma markers (7T dataset)

Fasted EDTA plasma samples were obtained through venipuncture from the antecubital vein and processed according to the SOP stipulated by the central biobank of Maastricht University Medical Center. Samples were centrifuged at 2000 × *g*, aliquoted in polypropylene tubes, and stored at −80 °C in our biobank within 60 min of collection. Plasma biomarkers were analyzed in randomized order using ultra-sensitive Single molecule array (Simoa) assays (Quanterix, Inc) for Aβ_42_ and Aβ_40_ (to create Aβ_42/40_ ratio), total tau (Neurology 3-Plex A Advantage Kit), ptau_181_ (pTau-181 V2 Advantage Kit), ptau_231_ (University Gothenburg) [28] and NfL (NF-light™ Advantage Kit) at the University of Gothenburg (Sweden). Analyses were performed in duplicates using a 1:4 automated dilution protocol for all markers, except for 1:2 dilution protocol for ptau_231_. Analysis of plasma ptau_217_ was performed at Lund University (Sweden) using the Meso Scale Discovery (MSD) platform as previously described [24]. The range of values measured is consistent with other studies examining similar cohorts (Table accompanying Supplemental Fig. 3). Based on a previously defined cut-off (Aβ_42/40_ ratio=0.080 [30]) in an asymptomatic cohort using the same assay in the same lab (University Gothenburg) we identified 20 Aβ + individuals. *APOE* genotyping was performed using polymerase chain reaction based on blood sample DNA extraction. Participants’ *APOE* status was defined as ‘ε4 carrier’ if they carry at least one ε4 allele. 48.5% (*n* = 48) carried at least one ε4 allele and/or has elevated plasma Aβ_42/40_ values, and hence can be considered at-risk of developing AD. Technicians handling the blood samples were blinded to the participant, cognitive and imaging data, and staff collecting cognitive or imaging data were blinded to blood results.

### Neuropathological measures (MAP dataset)

Immediately after participants’ death, brains were extracted, weighed, and the brainstem and cerebellar hemispheres removed. Both hemispheres and the brainstem were sectioned into 1 cm-thick coronal slabs. One hemisphere was frozen as were select samples of the brainstem; the remained was fixed in 4% paraformaldehyde. The average postmortem time was 7.09 h (SD:3.89 h). Neuronal density (per mm^2^) and paired helical filaments (PHF) tau tangle density of the LC were examined using immunohistochemistry with a monoclonal anti-tyrosine hydroxylase antibody and an anti-paired helical filaments tau antibody AT8, respectively, each bilaterally at two levels of the LC (rostral-to-middle (“rostral”) and main body or middle-to-caudal (“caudal”)) [42, 43]. To allow for unbiased comparisons, we divided tangle density by the neuronal density for each section of the LC. We selected participants who had neuropathologic data on both sections of the LC (*n* = 77).

Cortical Aβ load was quantified as percent area occupied by Aβ, labeled with a N-terminal directed monoclonal antibody, which identifies both the 1–40 and 1–42 length Aβ fragments, while cortical PHF tau tangles, were quantified as the density of paired helical filament tau tangles with an antibody specific for phosphorylated tau, AT8 (density per square millimeter) across 8 regions [12]. Modified Bielschowsky silver quantification was used for Braak scoring of neurofibrillary pathology and Consortium to Establish a Registry for AD (CERAD) scoring of neuritic plaques. Using this information, the likelihood of AD pathology was identified according to the modified National Institute of Aging (NIA)-Reagan diagnosis of AD and grouped into not present (no or low likelihood) and present (intermediate and high likelihood). This evaluation is performed independent of clinical information, including the diagnosis [37, 44].

### Neuropsychological assessment (7T dataset)

The preclinical Alzheimer’s cognitive composite (PACC) was designed to be sensitive to cognitive change among individuals with preclinical AD, and consists of the average of z-transformed scores on the Mini-Mental-State Examination, Logical Memory Delayed recall test, Digit Symbol Substitution Test, Free and Cued Selective Reminding test (free and total recall) and was later expanded to also include the category fluency [45]. We created a PACC-score based on the average of the z-scores of the performance on cognitive tests available in this cohort: Mini-Mental-State Examination, Digit Symbol Substitution Test, Rey-Auditory Verbal Learning Test (total and delayed free recall) and the category fluency test. Other collected behavioral measures were not analyzed for this study.

### Statistical analyses

Statistical analyses were performed in R (version 4.1.2, http://www.r-project.org/). Group characteristics are represented in mean and standard deviation. Associations between the plasma markers, and age, sex, or APOE-status were examined with robust regression using the Huber M estimator. Robust regression is suited to handle outliers that are often observed in the skewed distributions of biomarker data. It effectively downweighs their influence on the coefficients and regression fit and therefore, allows including individuals with more extreme (or abnormal) values, who are less prevalent in healthy populations but reflect the normal population. Voxel-wise robust regressions between LC intensity and each plasma marker, or their interactive or additive effects with plasma Aβ_42/40_ were corrected for age, sex and *APOE-*ε4 status and adjusted for multiple testing using the probabilistic Threshold Free Cluster Enhancement at two-sided *p* < 0.001 per analysis (not across analyses). From each plasma marker cluster on the LC surface, we extracted subject-specific LC intensity values for further analyses. These plasma-based analyses were repeated for the entire LC intensity, bilateral LC volume, and hippocampal volume to examine the specificity of our findings. To examine whether associations between the plasma markers and LC intensity in their respective clusters occurred within a specific age-range, we ran sliding window analyses with 20-year age bins with bootstrapped 95% confidence intervals (5,000 iterations) [46]. Finally, using robust regression we examined whether the plasma markers that were significant from the previous analyses and the extracted LC intensity from the plasma-specific clusters were interactively or independently associated with PACC performance, with age, sex, education and *APOE-*ε4 status as covariates. We adjusted here for multiple testing using Bonferroni correction. Using the Johnson-Neyman approach we determined at which value the plasma marker modified the relationship between LC intensity and PACC. The threshold for statistical significance was set at two-sided *p* < 0.05, unless otherwise specified.

For the MAP dataset, we performed a repeated measures ANOVA with proportion of tangles in each section of the LC as within-subject factor, and age, sex, *APOE-*ε4 status, and postmortem interval as covariates. To test if these effects are independent of AD pathology, we also added cortical Aβ or NIA-Reagan diagnosis of AD groups as covariate. In the second step, we included diagnosis (i.e., cognitively unimpaired, MCI or AD) as between-subject factor and interactions with LC sections, to determine whether the regional distribution of the proportion of LC tangles varied as a function of disease stage. Post-hoc adjustment for multiple comparisons was performed with Tukey’s HSD.

## Results

### Participant characteristics

In the 7 T dataset, the mean age of the participants was 59.93 years (range 30–85 years), 52 were female (52.50%) and 37 (37%) carried at least one *APOE-*ε4 allele (Table 1). All participants were cognitively healthy (mean MMSE-score:28.98). In the MAP sample, the mean age of the participants was 88.59 years, 56 were female (72.72%), 10 carried at least one *APOE-*ε4 allele (13%), and 47 individuals (61%) showed evidence of AD pathology at autopsy according to the NIA-Reagan AD criteria. Histograms depicting age distributions of each cohort are provided in Supplemental Fig. 2

**Table 1:** Participant characteristics.

	7 T dataset (*N* = 99)		MAP-study (*N* = 77)
Age (years)	59.93 (13.13)	Age (years)	88.59 (5.84)
Female (n, %)	52 (52.50%)	Female (n, %)	56 (72.72%)
Education (years)	14.44 (2.14)	Education (years)	14.44 (2.66)
MMSE (score)	28.98 (1.13)	MMSE (score)	23.52 (6.93)^$^
PACC (score)Range:	0.001 (0.67) −1.84–1.53		
*APOE* ε4 (n, %)	37 (37.37%)	APOE-ε4 (n, %)	10 (12.99%)
		Diagnosis (n, %)	29 CN (37.66%) 27 MCI (35.06%) 21 AD (27.27%)
		AD present (NIA-Reagan AD) (n, %)	47 (61.04%)
Aβ_42/40_	0.09 (0.01)	Cortical Aβ	4.82 (4.58)
ptau_181_ (pg/ml)	1.67 (0.72)	Cortical tangles	5.35 (5.34)
ptau_217_ (pg/ml)	0.27 (0.11)		
ptau_231_ (pg/ml)	8.55 (3.82)		
Total tau (pg/ml)	2.65 (0.89)		
NfL (pg/ml)	18.28 (7.05)		
Adjusted hippocampus volume (mm^3^)	6523.63 (673.76)		
LC intensity (a.u.)^#^	0.15 (0.06)	Relative LC tangles^#^	0.12 (0.14)

Demographics are presented in mean, standard deviation, or proportion. $:missing data for *n* = 5; #: these values are based on the entire LC.

*MMSE* Mini-Mental State Examination, *PACC* Preclinical Alzheimer’s disease cognitive composite, *CN* cognitively normal, *MCI* mild cognitive impairment.

### Correlations of plasma biomarkers with demographics

**Fig. 1:**
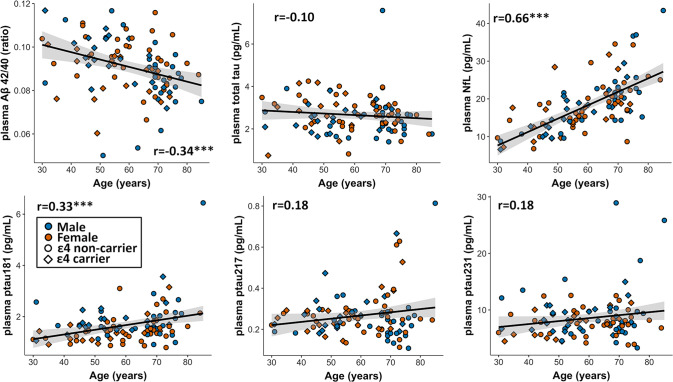
Age-associations with the plasma biomarkers. Note: Correlations reflect the relationship between age and the plasma marker for the entire cohort. Males are indicated in blue, females in orange, ε4 non-carriers in circles and ε4 carriers in diamonds. Shaded region shows the 95% confidence interval of the regression fit (see also Supplementary Tables 1, 2 for all correlations in the entire sample and participants >50 years).

First-order correlations between all the plasma markers, and age, sex or APOE are shown in Supplementary Tables 1, 2 and Supplemental Fig. 3. In the following analyses, age analyses were adjusted for sex, and the sex analyses were adjusted for age. Older age was associated with lower Aβ_42/40_ (t = −4.63, *p* < 0.001) and higher ptau_181_ (t = 3.25, *p* = 0.002) and NfL (t = 9.67, *p* < 0.001). We detected no age-relationship for total tau (t = −1.73, *p* = 0.08), ptau_217_ (t = 0.40, *p* = 0.69) or ptau_231_ (t = 1.17, *p* = 0.25). Very weak or a lack of significant correlations between the ptau markers and Aβ_42/40_ has been reported in similar cohorts [30]. Females exhibited higher total tau (t = −2.23, *p* = 0.023), while males displayed higher ptau_181_ (t = 3.21, *p* = 0.002). These sex differences remained significant when controlling for *APOE-*ε4 status and Aβ_42/40_ (t = −2.22, *p* = 0.03 and t = 3.82, *p* = 0.003, respectively). There were no sex differences in Aβ_42_/_40_ (t = 0.22, *p* = 0.83), NfL (t = 0.47, *p* = 0.64), ptau_217_ (t = −0.81, *p* = 0.43) or ptau_231_ (t = 1.47, *p* = 0.15). Those who carry at least one ε4 allele displayed lower Aβ_42/40_ (t = −2.27, *p* = 0.025). We observed no differences between ε4-carriers and non-carriers for other biomarkers (total tau: *p* = 0.79, NfL: *p* = 0.95, ptau_181_: *p* = 0.18, ptau_217_: *p* = 0.15, ptau_231_: *p* = 0.66, Fig. 1).

### Voxel-wise relationships between locus coeruleus intensity and plasma biomarkers

**Fig. 2:**
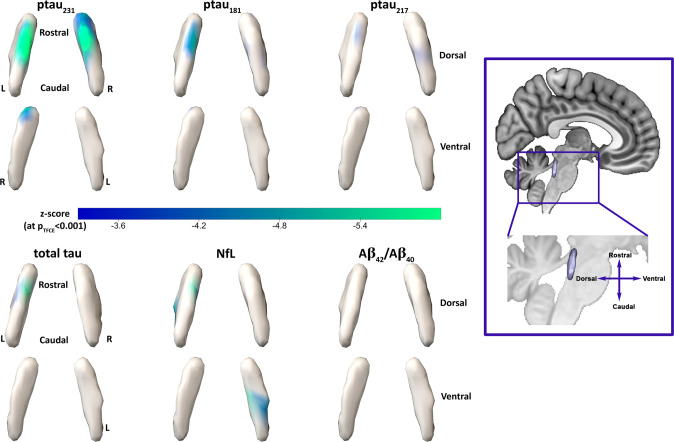
Voxel-wise associations between LC intensity and the plasma markers. Note: Analyses were adjusted for age, sex, *APOE* ε4 status and multiple comparisons using the threshold-free cluster enhancement approach (TFCE) at *p* < 0.001. Color scale shows the corresponding z-scores. The box on the right provides information for anatomical orientation of the LC surfaces.L = left, R-right.

Age, sex and *APOE-*ε4 status were included as covariates in the voxel-wise regression analyses, given their significant associations with the plasma biomarkers. Voxel-wise analyses revealed that higher ptau_231_ was associated with lower LC intensity in bilateral dorso-rostral clusters (p_TFCE_ <0.001). Ptau_231_ had the largest cluster-size (160 voxels). Ptau_181_ (44 voxels), ptau_217_ (37 voxels) and t-tau (49 voxels) correlated negatively with LC intensity in right dorso-rostral clusters. Small clusters of negative associations between LC intensity and NfL (51 voxels) were distributed across the length of the LC, and more prominent in the middle-to-caudal part. LC intensity was not associated with Aβ_42/40_ (Fig. 2). Adjusting for Aβ_42/40_ did not change these associations. We did not find interactions between Aβ_42/40_ and any of the other plasma markers on LC intensity. Consistent with this, we observed similar negative slopes between the ptau markers, in particular the ptau_231_, and intensity in the LC clusters across Aβ + and Aβ- groups (Supplemental Fig. 4). The control brain measures, hippocampal volume, bilateral LC volume, and average entire LC intensity, were not associated with any of the plasma markers (Supplemental Table 3).

### Age-window of the relationship between locus coeruleus intensity and plasma biomarkers

**Fig. 3:**
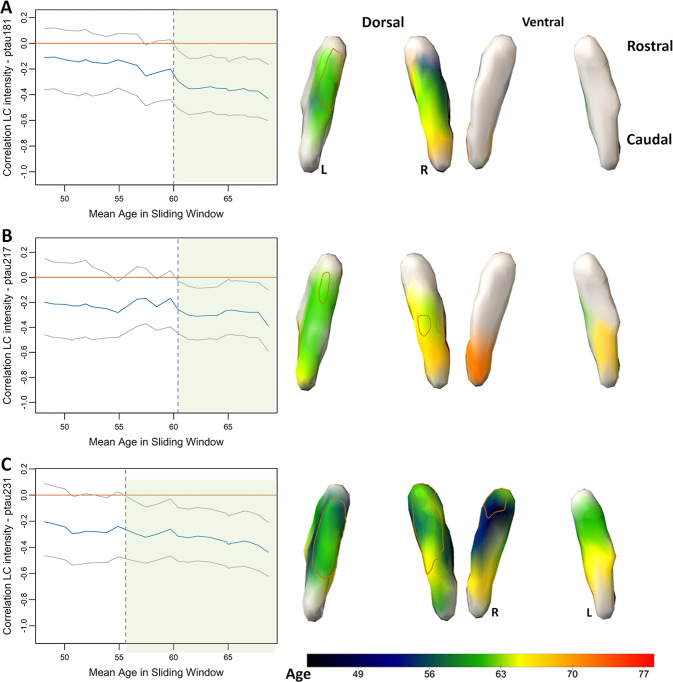
Age-windows of associations between plasma markers and LC intensity. Note: Results of the bootstrapped sliding window analyses: blue line depicts the correlation coefficient between the plasma marker and the respective LC intensity cluster, the gray lines indicate the bootstrapped 95% confidence interval (5000 replicates at *p* < 0.05). Age window where the association was robustly significant is marked in the green shaded region with the starting age indicated by the dashed line. **A** Results for plasma ptau_181_, (**B**) ptau_217_ and (**C**) ptau_231_. To map the anatomy of these associations, we also performed bootstrapped voxel-wise sliding window analyses examining the age-window of associations between regional LC intensity and each ptau marker (right column). The scale bar shows the age window where the associations were observed (cold (blue)): starting at younger ages; warm (orange): starting at older ages. White regions on the surface indicate voxels where no robust age-window was detected. The orange demarcation on the LC surfaces show the significant clusters observed in Fig. 2.

Bootstrapped sliding window analyses showed that the relationship between ptau_181_ and LC intensity in its cluster was significant for individuals older than 60 (Fig. 3A), for ptau_217_ starting from 60.5 years (Fig. 3B), and for ptau_231_ the relationship with LC intensity was significant from 55.5 years and older (Fig. 3C). Of the individuals older than 55.5 years, 41% (25/61) was considered at increased risk for AD-related processes based on Aβ < 0.08 or presence of at least one *APOE* ε4 allele. For NfL and total tau, no robust age windows were detected. We confirmed these patterns in voxel-wise sliding window analyses: associations between ptau_181_ and rostral dorsal LC intensity emerged in the early 60 s, while for ptau_217_, we detected clusters in the dorsal rostro-middle LC from the early-mid 60 s. The earliest age-associations with LC intensity were found for ptau_231_, revealing an anatomic pattern in the ventral and dorsal rostral parts of the LC starting from late 40 s and later associations (60’s) in the middle-caudal section of the LC (Fig. 3A–C).

### Independent and interactive relationships of plasma biomarkers and LC intensity on PACC

**Fig. 4:**
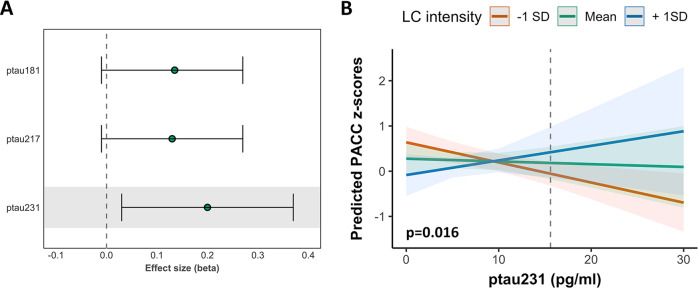
Interactive relationships between LC intensity and plasma markers in predicting PACC performance. Note: **A** effect sizes and 95% confidence intervals of the interaction between each biomarker and the respective LC intensity cluster on PACC performance. The shaded region indicates the best model (ptau_231_) and this association is depicted in (**B**). **B** At values above ~15.7 pg/ml of ptau_231_, lower LC integrity is associated with lower PACC score.

Based on the previous results, we focused the cognitive analyses on the ptau biomarkers. The interaction between ptau_181_ and its associated LC intensity cluster on PACC performance was significant at trend-level (*p* = 0.06). There were no independent effects of ptau_181_ and LC intensity on PACC. We found no interactive or independent relationships of ptau_217_ with LC intensity in predicting PACC performance (Supplemental Table 4). At higher levels of ptau_231_, in particular above 15.69 pg/ml, lower LC intensity was associated with worse PACC performance (*p* = 0.016 or p_BONF_ = 0.048, Fig. 4).

### Regional specificity of tau in the LC in the autopsy dataset

**Fig. 5:**
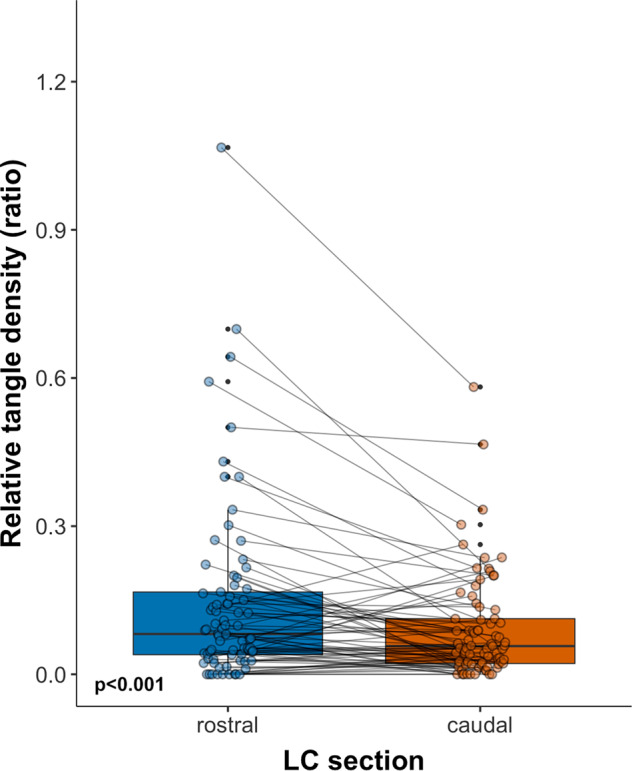
Rostral versus caudal tangle density in the LC in the MAP dataset. Note: Boxplot depicting the rostral and caudal tangle LC density (relative to its neuronal density) for the entire MAP sample (MAP dataset, *N* = 77). The boxplot depicts the interquartile interval (quartile one to three), with the horizontal line in the box representing the median.

In the MAP dataset, normalized density of LC tangles in the rostral LC was significantly greater than those in the caudal LC (F_(1,72)_ = 13.14, *p* < 0.001). This difference remained present when controlling for cortical Aβ (F_(1,71)_=13.39,*p* < 0.001) or NIA-Reagan diagnosis of AD groups (F_(1,71)_ = 12.96, *p* < 0.001, Fig. 5).

Upon examining the interaction between LC section and the diagnostic groups, we found no significant difference in rostral versus caudal LC tangle density across the groups (F_(1,70)_ = 0.07, *p* = 0.94, Supplemental Fig. 5). Across all groups, rostral LC tangle density was higher than caudal LC tangle density (contrast of main effect LC section:β=0.50, t = 2.21, *p* = 0.031; mean rostro-caudal LC tangle density difference for cognitively normal = 0.05 ± 0.13, for MCI = 0.05 ± 0.09 and for AD = 0.05 ± 0.15).

## Discussion

As clinical trials targeting Aβ provided marginal clinical effects, the AD field has oriented its focus on earlier time windows in the disease process [3]. These earlier windows have compelled researchers to focus on tau pathology, which is more closely related to the emergence of clinical symptoms compared to Aβ [47, 48]. The LC is one of the earliest brain regions accumulating hyperphosphorylated tau [6] and recent work emphasized its potential as early marker of future AD-related processes, including tau and cognitive decline [11, 12, 17]. Here we aimed to examine whether different tau species and markers of neurodegenerations correlate to specific anatomic patterns of integrity within the LC across the adult lifespan. We found that lower integrity in bilateral dorso-rostral clusters of the LC was associated with greater ptau_231_ concentrations, starting from midlife (~55 years). Higher ptau_217_ and ptau_181_ levels were associated with lower LC integrity in smaller right dorso-rostral clusters, starting from age 60. Similarly, the autopsy data revealed a higher tangle density in the rostral compared to the caudal part of the LC, independent of AD likelihood. Furthermore, lower PACC scores were associated with lower rostral LC integrity, particularly for individuals with higher plasma ptau_231_. By contrast, higher NfL was associated with predominantly lower middle-to-caudle LC integrity, independent of age. As tau phosphorylation at threonine 231 is one of the earliest events in the phosphorylation cascade hindering tubulin assembly [49], these findings illustrate that changes in rostral LC intensity can reflect processes related to very early tau aggregation, whereas integrity changes towards the caudal direction in the LC may reflect more nonspecific neurodegeneration.

The fine-grained topography of correlations between LC integrity and ptau markers, demonstrating a predilection of AD-related processes for rostral regions of the LC, is consistent with autopsy reports [13, 50]. Work by Theofilas and colleagues reported 8.40% volume loss – not neuronal loss – per Braak stage, starting in Braak stage 0 for the rostral and middle LC [13]. Consistent with our previous work [32] and with the observation that the caudal LC contains fewer AD-related changes [13], we found that indicators of nonspecific neurodegeneration were associated with lower caudal LC integrity. Ultra-high field imaging of the LC in Parkinson’s disease reported lower integrity in the caudal part in patients compared to controls [51]. We speculate that these observations emphasize a rosto-caudal gradient of vulnerabilities to specific pathologic events within the LC that may also be disease-specific. In accordance with this hypothesis, we found that rostral-middle parts of the LC are more vulnerable to hyperphosphorylated tau whereas the caudal part may be more affected by non-specific neurodegeneration.

With respect to the plasma ptau epitopes, we found that ptau_231_ was more robustly and earlier in life associated with LC integrity compared with the ptau_217_ and ptau_181_ markers. Neuropathology studies demonstrated that tau phosphorylation at threonine 231 may signal features of tau that precede the pre-neurofibrillary tangle [52] and reflect the earliest events in the phosphorylation cascade hindering tubulin assembly [49]. In the ALFA + cohort of asymptomatic individuals, ptau231 changes preceded changes in other markers and predicted increases in Aβ-PET burden among individuals with no PET-evidence of Aβ-pathology as well as in the younger group (45–60 years) [30]. Given the concordance between these spatial patterns in the voxel-wise analyses and the higher rostral vulnerability in the autopsy data, our previous report on converging patterns of associations between LC integrity and tau-PET with autopsy data [11], and the similarities between our cohort and the ALFA + cohort, our findings here support the idea that LC integrity measures may capture very early aberrant tau-related processes [6, 53, 54].

The finding that ptau_231_ may be an early marker fits with recent work showing that ptau_231_was able to differentiate between PET-derived Braak stage 0 and Braak stage I-II, whereas ptau_181_ was not [28]. Furthermore, elevations in ptau_231_ can be detected at lower amounts of AD-type pathology compared to ptau_181_ [55]. Importantly, similar observations have been made for cerebrospinal fluid ptau markers [56, 57]. Plasma ptau_181_ correlates well with tau-PET, can detect elevated Aβ-PET and discriminate AD patients from non-AD patients [25, 27]. Previous work demonstrated that plasma ptau_217_ detects AD-related processes slightly earlier than ptau_181_ as it was better in predicting elevated entorhinal tau [58]. We did not see a remarkable difference between the topography on the LC for ptau_217_ and ptau_181_. Furthermore, our sliding window analyses revealed a similar age-range (>60 years) during which both ptau_217_ and ptau_181_ have their strongest relationship with LC integrity. Our plasma Aβ_42/40_ marker did not correlate with the ptau markers, and this lack of correlations between the Aβ and ptau markers is consistent with observations in the ALFA + cohort [30]. Within the Aβ + and Aβ- groups, we observed similar associations between the ptau markers and LC integrity, in particular for ptau_231_, and we posit that these ptau_231_-related LC changes may capture early processes on the AD pathophysiologic continuum. Importantly, the sensitivity of detecting early tau-related LC changes is higher when honing in on the rostral LC instead of the entire average LC or LC volume. Furthermore, we did not observe tau-related associations with hippocampal volume, further emphasizing the specificity of the rostral LC as a potential marker to detect very early AD-related processes.

This rostro-caudal gradient of tau-related vulnerability within the LC also aligns with the topographic arrangement of axonal projections and the cytoarchitecture of the LC [14]. Projections of the LC are not randomly organized, as neurons in the rostro-middle sections of the LC preferentially project to the higher-order cognitive regions of the cortex, while the majority of the neurons in the caudal section of the LC projects to the cerebellum and spinal cord [59, 60]. Furthermore, the rostral and caudal parts of the LC have distinct cell types and cell sizes organized in clusters, supporting functional heterogeneity [61, 62]. The dorso-rostral LC contains almost uniquely densely packed small fusiform neurons, which have long, thin dendrites projecting to the hippocampus and cortex, and are more heavily pigmented and more vulnerable to pathology than the larger cells. The caudal part of the LC contains mostly large multipolar neurons [62–64]. This distribution may also support the rostral LC’s critical contribution in modulating cognition, because fusiform neurons tend to have shorter action potentials with larger amplitudes compared to multipolar cells [65, 66]. These electrophysiologic properties enable fusiform cells to sustain high frequency firing critical to focused attention, learning and responding to salient stimuli [61, 67].

Thus, the specific vulnerability of rostral neurons to hyperphosphorylated tau aggregation may affect the functioning of these smaller cells and hence, affect its capacity to modulate cognition. Notably, of all three ptau markers, ptau_231_ was also the only marker predicting PACC performance in interaction with rostral LC integrity, at a threshold value that was slightly higher than a recently published study reporting faster cognitive decline of unimpaired older individuals with elevated ptau_231_ [68]. Furthermore, in populations consisting of unimpaired and impaired individuals higher ptau_231_was associated with faster decline on the MMSE, Dementia Rating Scale and Clinical Dementia Rating-Sum of Boxes scale as compared to ptau_181_ [28, 55]. These findings further confirm that lower rostral LC integrity measures can signal risk for early AD-related processes.

This study has several limitations. First, while this is currently the largest cohort using state-of-the-art 7 T LC imaging, our sample size is moderately large. Due to the greater forces of the magnetic field, ultra-high field imaging has more strict inclusion criteria, resulting in lower enrollment and possibly a selection-bias to the healthier population. Second, due to the SARS-CoV-2 pandemic longitudinal assessments were delayed, limiting our current analyses to the cross-sectional data. Therefore, it also remains uncertain whether these individuals will exhibit progression consistent with AD trajectories or primary age-related tauopathy (PART) [69]. As we do not have topographical information of tau and because we cannot preclude disease progression given that this sample is still cognitively unimpaired, making a distinction between these pathways is difficult. The fact that ptau_231_ has shown to be a sensitive early marker of underlying AD pathology [28], predicts accumulation of Aβ [30], that almost half of the individuals >55.5 years in whom we found ptau-LC associations has either a genetic risk factor for AD or presence of elevated Aβ, and that these associations are also detected in the Aβ + individuals separately, makes it more likely that our observations reflect AD-related pathways. Nonetheless, follow-up of these individuals will be required to describe their phenotypic trajectory. Given that our neuropathologic observations confirm a rostral vulnerability independent of disease stage, similar associations can be expected in impaired individuals. Third, caution is needed when interpreting the caudal LC. As this region of the LC is more diffuse [70], it is also more challenging to image it and the data acquired may therefore not capture the full length of the LC. Therefore, volumetric LC measures should also be interpreted with caution. Finally, comparisons between the three ptau epitopes need to take into account that these markers were quantified using different platforms and antibodies [58].

To conclude, the plasma ptau_231_ marker is specifically associated with lower dorso-rostral LC integrity starting in midlife, and jointly, integrity of this LC section and plasma ptau_231_ predict lower PACC performance. These findings link a plasma marker presumed to reflect a very early phase of hyperphosphorylation of tau specifically to the part of the LC exhibiting early vulnerability to tau deposition. Reductions in integrity of the dorso-rostral LC may thus signal very early AD risk, including early tau aggregation.

### Supplementary information

Supplemental Materials

## Data Availability

Participants in the 7 T study did not explicitly consent to their data being made public. Requests for the anonymized data should be made to Dr. Heidi Jacobs (www.heidijacobs.org; h.jacobs@maastrichtuniversity.nl or hjacobs@mgh.harvard.edu) and will be reviewed by an independent data access committee, taking into account the research proposal and intended use of the data. Requestors are required to sign a data sharing agreement to ensure participants’ confidentiality is maintained prior to release of any data and that procedures conform with the EU legislation on the general data protection regulation and local ethical regulations. Data domains in which human data collection is ongoing can only be shared under these regulations once data collection and quality assessment are completed. Data from ROSMAP is available upon request at https://www.radc.rush.edu.
