# Supplementary material for: Ultra-high field imaging, plasma markers and autopsy data uncover a specific rostral locus coeruleus vulnerability to hyperphosphorylated tau
Source: Mol Psychiatry. 2023 Apr 5;28(6):2412–22. doi: 10.1038/s41380-023-02041-y (PMC10073793; doi:10.1038/s41380-023-02041-y)
Supplement: Supplementary file 1 — Supplemental Materials [file 41380_2023_2041_MOESM1_ESM.docx]

**Supplemental data:**

**Ultra-high field imaging, plasma markers and autopsy data uncover a specific rostral locus coeruleus vulnerability to hyperphosphorylated tau**

Maxime Van Egroo, Joost M Riphagen, Nicholas J Ashton, Shorena Janelidze, Reisa A Sperling, Keith A Johnson, Hyun-Sik Yang, David A. Bennett, Kaj Blennow, Oskar Hansson,

Henrik Zetterberg, Heidi IL Jacobs

**
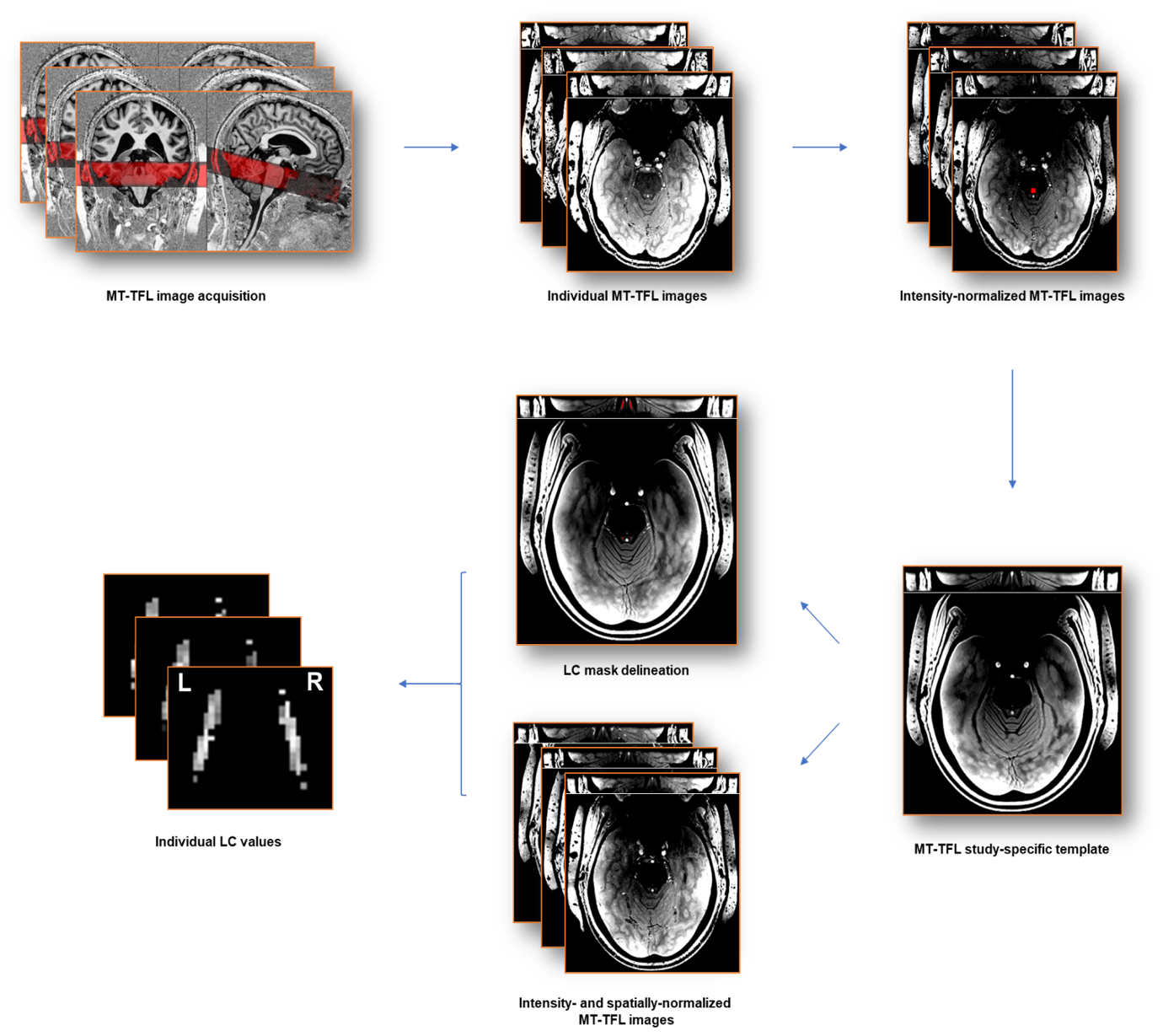
 Figure 1:** Overview of the 7T LC-imaging processing pipeline

Note: Our 7T LC MRI processing pipeline involved the following steps: first, individual MT-TFL images were intensity-normalized by dividing by the subject-specific mean intensity of a 10x10 voxel region-of-interest in the pontine tegmentum (PT). For each participant, the PT region-of-interest was consistently placed in the axial slice which contained the highest intensity LC voxel. As part of our quality check,, no association was found between age and mean intensity in the PT, supporting that no age-related biases would be introduced by this normalization step. Second, a study-specific template was built from these individual intensity-normalized MT-TFL images using the *buildtemplateparallel* function from the Advanced Normalization Tools (ANTs) (transformation model and similarity metric used for registration = greedy SyN with cross-correlation). The LC was then manually delineated on the resulting template, based on voxel intensities and the anatomical properties of the LC. The LC mask was then applied to each individual intensity-normalized MT-TFL images registered to the study-specific template. Either voxel-wise analyses were performed within these LC masks or average LC intensity values were extracted for each subject. The red square illustrates, for a representative participant, the 10x10 voxel region-of-interest located in the pontine tegmentum which was used for subject-specific normalization of MRI signal intensity (Reproduced from our previous work^1^).

**
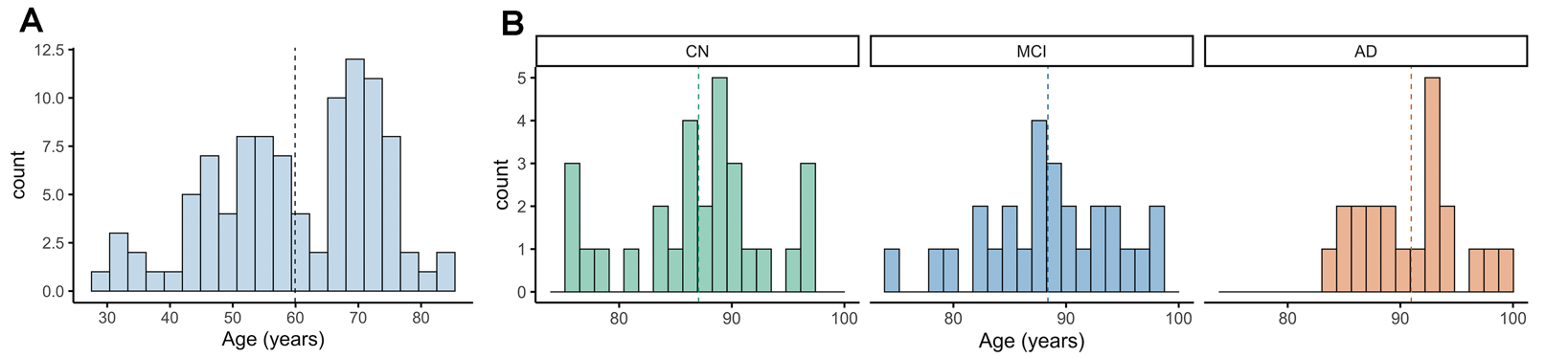
Figure 2:** Distribution of the age of participants across both cohorts and clinical diagnosis

**Note:** Histograms showing the distribution of the age of participants in the **A)** 7T dataset and **B)** in the MAP dataset, separated per clinical diagnosis. The dotted line indicates the mean age.

**Table 1:** First-order correlations between demographics and plasma biomarkers in the entire sample

|  | **Age** | **Sex** | **Educ** | **APOE** | **Aβ_42/40_** | **T-tau** | **NFL** | **ptau_181_** | **ptau_217_** |
| --- | --- | --- | --- | --- | --- | --- | --- | --- | --- |
| **Age** |  |  |  |  |  |  |  |  |  |
| **Sex** | 0.08 |  |  |  |  |  |  |  |  |
| **Educ** | -0.45  *** | -0.13 |  |  |  |  |  |  |  |
| **APOE** | -0.33  *** | -0.10 | 0.23  * |  |  |  |  |  |  |
| **Aβ_42/40_** | -0.34  *** | 0.05 | 0.11 | -0.03 |  |  |  |  |  |
| **T-Tau** | -0.11 | 0.11 | 0.01 | -0.01 | -0.00 |  |  |  |  |
| **NFL** | 0.66  *** | -0.06 | -0.25  * | -0.19 | -0.25  * | -0.01 |  |  |  |
| **ptau_181_** | 0.33  *** | -0.27  ** | -0.06 | -0.01 | -0.12 | -0.02 | 0.43  *** |  |  |
| **ptau_217_** | 0.18  ^#^ | 0.02 | 0.02 | 0.08 | 0.05 | -0.11 | 0.28  ** | 0.60  *** |  |
| **ptau_231_** | 0.18  ^#^ | -0.22  * | -0.01 | -0.07 | -0.03 | 0.50  *** | 0.30  ** | 0.74  *** | 0.32  ** |

Note: Cells contain the Pearson product-moment correlation coefficients when correlating two continuous variables or the point-biserial when there is a dichotomous variable. Abbreviations: Educ: Education, *:p<0.05, **:p<0.01, ***:p<0.001,^#^: p<0.10

**Table 2:** First-order correlations between demographics and plasma biomarkers in individuals >50 years (n=75)

|  | **Age** | **Sex** | **Educ** | **APOE** | **Aβ_42/40_** | **T-tau** | **NFL** | **ptau_181_** | **ptau_217_** |
| --- | --- | --- | --- | --- | --- | --- | --- | --- | --- |
| **Age** |  |  |  |  |  |  |  |  |  |
| **Sex** | 0.05 |  |  |  |  |  |  |  |  |
| **Educ** | -0.33  ** | 0.22  ^#^ |  |  |  |  |  |  |  |
| **APOE** | -0.27  * | 0.07 | 0.17 |  |  |  |  |  |  |
| **Aβ_42/40_** | -0.33  ** | -0.11 | 0.06 | -0.18 |  |  |  |  |  |
| **T-Tau** | -0.06 | -0.03 | 0.004 | 0.05 | 0.03 |  |  |  |  |
| **NFL** | 0.57  *** | 0.12 | -0.12 | -0.10 | -0.21  ^#^ | 0.05 |  |  |  |
| **ptau_181_** | 0.34  ** | 0.21  ^#^ | 0.05 | 0.06 | -0.11 | 0.01 | 0.48  *** |  |  |
| **ptau_217_** | 0.20  ^#^ | -0.04 | 0.05 | 0.10 | -0.04 | -0.07 | 0.29  * | 0.66  *** |  |
| **ptau_231_** | 0.21  ^#^ | 0.18 | 0.09 | 0.002 | -0.04 | 0.51  *** | 0.38  *** | 0.76  *** | 0.39  *** |

Note: Cells contain the Pearson product-moment correlation coefficients when correlating two continuous variables or the point-biserial when there is a dichotomous variable. Abbreviations: Educ: Education, *:p<0.05, **:p<0.01, ***:p<0.001,^#^: p<0.10


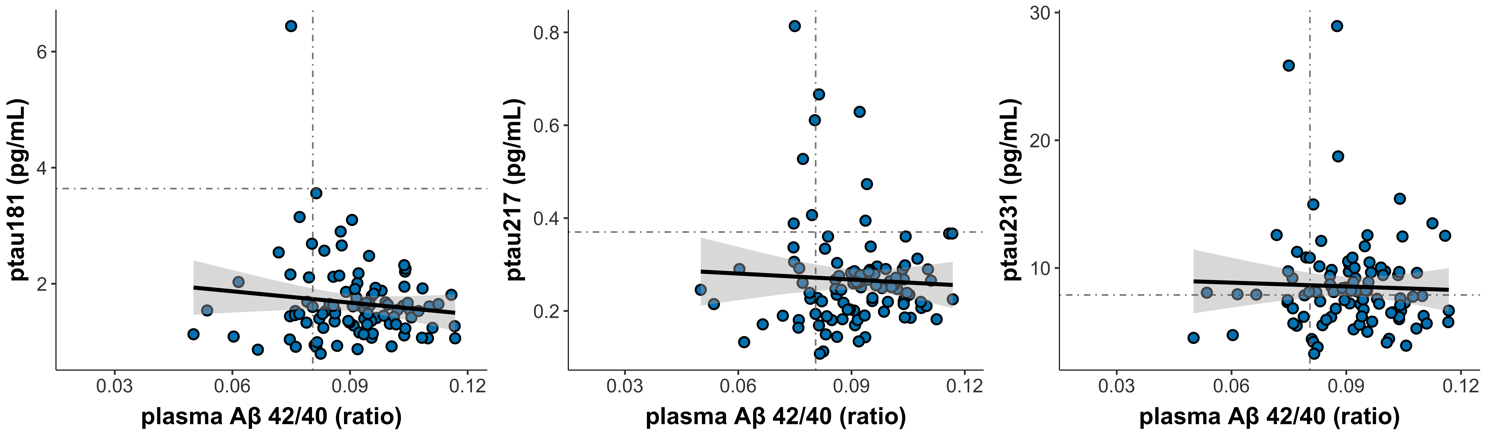
**Figure 3:** Correlations between plasma ptau markers and plasma Aβ_42/40_

Note: Scatterplots show the correlations between plasma Aβ42/40 and the three ptau markers. There were no significant relationships between the markers. The dotted lines indicate cut-offs from studies closest to our work using the same assays and if possible, performed in the same lab: for Aβ_42/40_ (0.08) and ptau_231_ (7.91 pg/mL) ^2^; for ptau_181_ (3.962 pg/mL): ^3^; for ptau_217_ (0.37 pg/mL):^4^; for comparisons the ranges of the obtained samples in these studies is provided in the below table, along with the range in our sample).

|  | Estimated range in literature | Range in our sample |
| --- | --- | --- |
| Aβ_42/40_ | [0.03 – 0.13]^a^ | [0.05 – 0.12] |
| ptau_231_ | [2.00– 39.00]^b^ | [3.29 – 28.94] |
| ptau_181_ | [0.00 – 9.00]^c^ | [0.80 – 6.44] |
| ptau_217_ | [0.00 - 0.95]^d^ | [0.11 – 0.81] |

Note: ^a:^ Estimated from the violin plots of Suppl. Fig 1 (all participants, with and without biomarker evidence). Cut-off was derived from Suppl Table 5 (sensitivity 85%:^2^). ^b^: Estimated from the violin plots of Suppl. Fig 1 (all participants, with and without biomarker evidence). Cut-off was derived from Suppl Table 5 (sensitivity 85%^2^). ^c^: Given the high proportion of MCI/AD patients in the high ADNC group, we derived the range from the boxplot in Figur1 (all participants in the low and intermediate ADNC, which also included a lower number of impaired individuals). The threshold was identified in the ROC analyses comparing low to high ADNC (Figure 4^3^) ^d^: Estimated from the boxplots in Figure 2A (identified threshold represents the robust normed^4^).


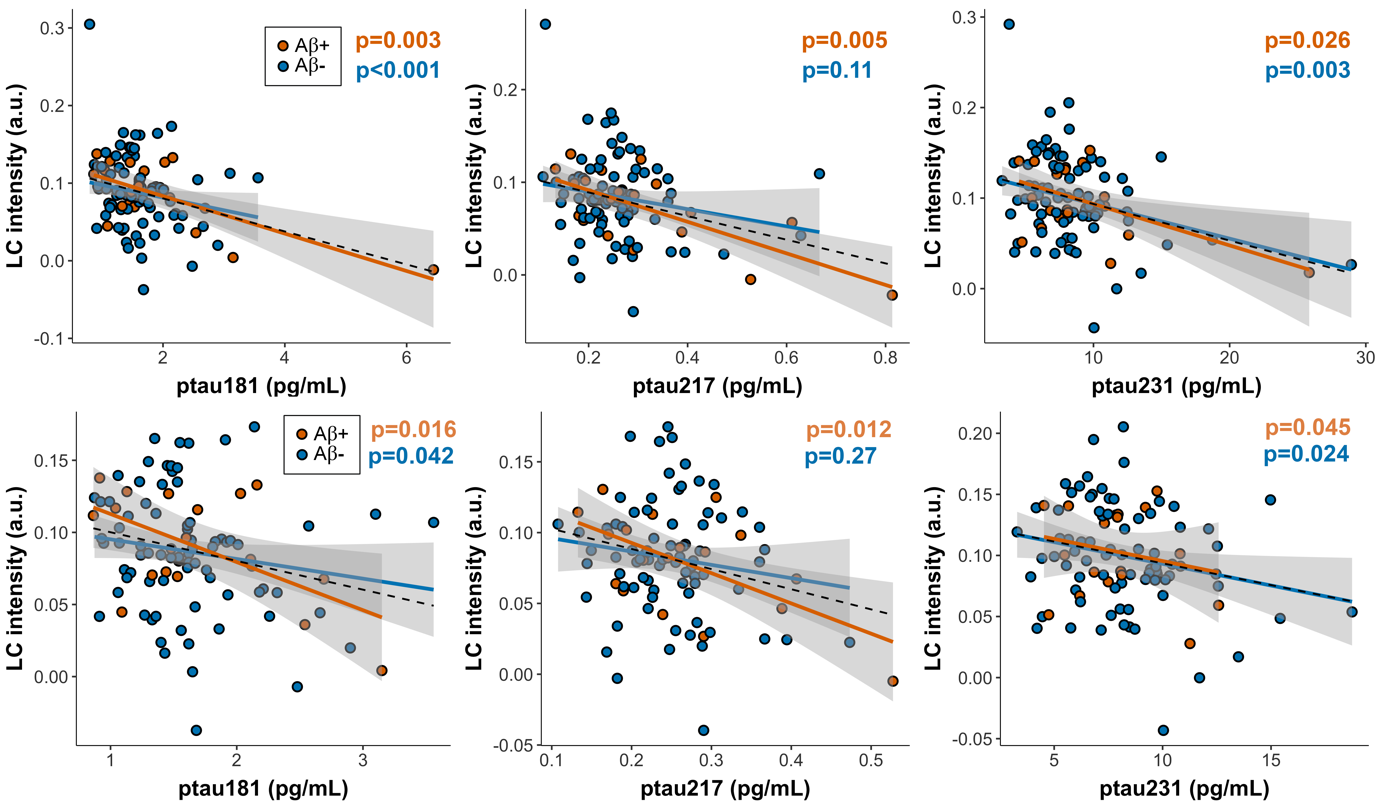
**Figure 4:** Associations between ptau markers and clusters of LC intensity among Aβ+ or Aβ- individuals

**Note:** The datapoints and regression fit demonstrates the association between each ptau marker and LC intensity in the detected cluster (Figure 2) within the Aβ- (blue, n=79) or Aβ+ (orange, n=20) group. Associations were tested with a robust regression (using Huber-M estimator) to account for outliers and influential points and adjusted for age and sex. To ensure that the weighing algorithm of the robust regression accounted for the observed outlier(s), we reran the analyses excluding the outliers and effects remained similar within the two groups (bottom row). Results for whole group analyses: ptau_181_ (Aβ+: n=19; Aβ-: n=78; entire sample: p=0.004), for ptau_217_ (Aβ+: n=18,Aβ-; n=76; entire sample: p=0.017), for ptau_231_ (Aβ+: n=19; Aβ-: n=77; entire sample: p=0.011).

**Table 3:** Control analyses with the plasma biomarkers

|  | **Beta** | **t-value** | **p-value** |
| --- | --- | --- | --- |
| **Average entire LC intensity** | | | |
| **Aβ_42/40_** | -0.25 | -0.67 | 0.50 |
| **T-Tau** | -0.01 | -0.95 | 0.34 |
| **NFL** | -0.002 | -1.24 | 0.24 |
| **ptau_181_** | -0.01 | -1.69 | 0.10 |
| **ptau_217_** | -0.05 | -1.09 | 0.28 |
| **ptau_231_** | -0.01 | -1.76 | 0.08 |
| **Bilateral LC volume** | | | |
| **Aβ_42/40_** | 90.99 | 0.76 | 0.44 |
| **T-Tau** | 0.33 | 0.20 | 0.84 |
| **NFL** | -0.15 | -0.53 | 0.59 |
| **ptau_181_** | -1.10 | -0.50 | 0.61 |
| **ptau_217_** | 1.49 | 0.11 | 0.91 |
| **ptau_231_** | 0.04 | 0.08 | 0.93 |
| **Adjusted hippocampus volume** | | | |
| **Aβ_42/40_** | 4017.04 | 0.84 | 0.40 |
| **T-Tau** | 63.24 | 0.96 | 0.33 |
| **NFL** | -13.90 | -1.23 | 0.22 |
| **ptau_181_** | -132.79 | -1.49 | 0.14 |
| **ptau_217_** | -945.79 | -1.80 | 0.08 |
| **ptau_231_** | -11.20 | -0.68 | 0.52 |

Note: Results of the robust regression associating overall average LC intensity, bilateral LC volume or hippocampal volume to the plasma markers, including age, sex, and APOE-status as covariates. Beta’s are the unstandardized coefficients. Abbreviations: LC = locus coeruleus, NfL = neurofilament light.

**Table 4:** Interactive versus independent relationships between LC intensity and plasma markers on PACC performance

|  | **Beta** | **t-value** | **p-value** |
| --- | --- | --- | --- |
| **ptau_181_* LC intensity** | 2.64 | 1.90 | 0.06 |
| **ptau_181_** | -0.18 | -1.75 | 0.08 |
| **LC intensity** | -1.61 | -1.12 | 0.27 |
| **ptau_217_* LC intensity** | 17.59 | 1.85 | 0.06 |
| **ptau_217_** | -0.41 | -0.67 | 0.51 |
| **LC intensity** | 1.37 | -0.92 | 0.37 |
| **ptau_231_* LC intensity** | 0.77 | 2.40 | 0.016  (BONF: p=0.048) |

Note: Results of the interactive models between LC intensity and the plasma markers in predicting PACC performance. When the interaction was not significantly, we examined potential independent effects (right-justified). Robust regressions with age, sex, education as covariates. Beta’s are the unstandardized coefficients. Abbreviations: LC: locus coeruleus, BONF = Bonferroni correction for multiple testing.

**Figure 5:** Rostral versus caudal LC tangle density across diagnostic groups


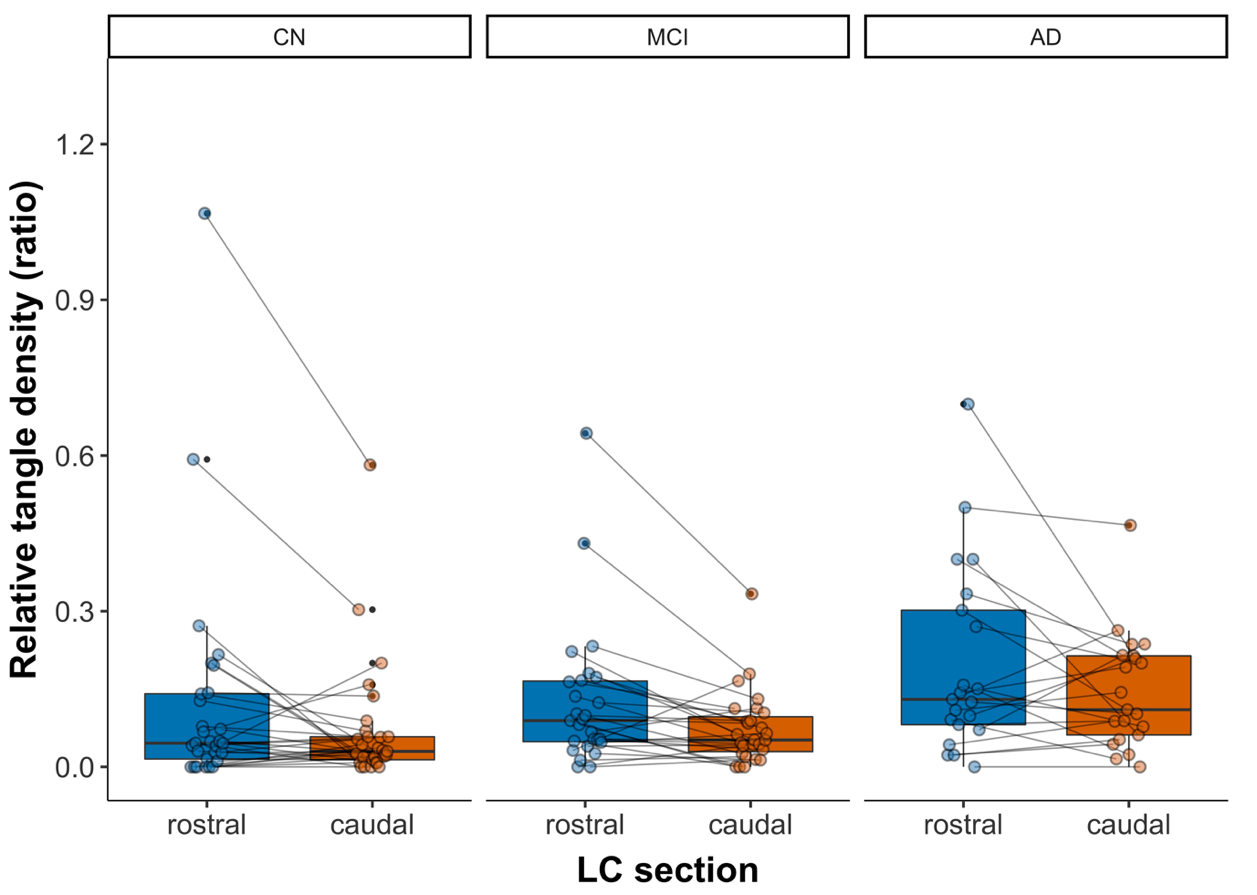


Note: Boxplot depicting similar rostral versus caudal tangle LC density (relative to its neuronal density) differences across the three groups (cognitively normal, MCI and AD patients). The boxplot depicts the interquartile interval (quartile one to three), with the horizontal line in the box representing the median.

**References**

1. Van Egroo M, van Hooren RWE, Jacobs HIL. Associations between locus coeruleus integrity and nocturnal awakenings in the context of Alzheimer's disease plasma biomarkers: a 7T MRI study. *Alzheimers Res Ther* 2021; **13**(1)**:** 159.

2. Mila-Aloma M, Ashton NJ, Shekari M, Salvado G, Ortiz-Romero P, Montoliu-Gaya L *et al.* Plasma p-tau231 and p-tau217 as state markers of amyloid-beta pathology in preclinical Alzheimer's disease. *Nature medicine* 2022.

3. Smirnov DS, Ashton NJ, Blennow K, Zetterberg H, Simren J, Lantero-Rodriguez J *et al.* Plasma biomarkers for Alzheimer's Disease in relation to neuropathology and cognitive change. *Acta neuropathologica* 2022.

4. Jonaitis EM, Janelidze S, Cody KA, Langhough K, Du L, Chin NA *et al.* Plasma pTau-217 in preclinical Alzheimer’s disease. *medRxiv* 2022.
